# A Physical Activity Intervention for High-Risk Mothers Whose Children Play Sports: Qualitative Analysis of Exit Interviews From Two Pilot Studies

**DOI:** 10.2196/87120

**Published:** 2026-10-08

**Authors:** Sugandha K Gupta-Louis, Belinda O'Hagan, Naomi Raisman, Gabriel Prieto, Michael C Onu, Bess H Marcus, Tayla von Ash

**Affiliations:** 1 Ferkauf Graduate School of Psychology, Yeshiva University, Rousso Building, Room 232, 1165 Morris Park Ave, Bronx, NY, 10461, United States, 1 646-592-4519; 2 Brown University School of Public Health, Providence, RI, United States; 3 Brown University, Providence, RI, United States

**Keywords:** community-based research, qualitative research, intervention study, program evaluation, gender, exercise, physical activity

## Abstract

**Background:**

Mothers, especially those with low socioeconomic status and minoritized identities, encounter barriers to participating in physical activity (PA), a behavior that improves health outcomes. Moms on the Move (MOMs) is a community-based and theory-informed intervention developed to address the PA barriers endorsed by low-income Black and Hispanic mothers. This intervention offered in-person group PA sessions led by a female fitness instructor and held during children’s sports practices. The MOMs intervention was piloted in 2022 and 2023; the intervention in 2023 was longer (8-weeks, vs 6-weeks) and included goal-setting calls and written materials.

**Objective:**

This study examined the qualitative data from exit interviews of both pilot studies to explore participants’ engagement and satisfaction with the intervention. We also sought to identify perceived benefits and barriers to participation, in addition to recommendations for future interventions.

**Methods:**

Exit interviews of 19 participants who had enrolled in at least 1 MOMs pilot study were analyzed with directed content analysis. Interviews were approximately 16 minutes on average. Social Cognitive Theory (SCT) and the Transtheoretical Model (TTM) were incorporated as theoretical frameworks for data coding and analysis.

**Results:**

Around half the participants identified as Black or Hispanic and reported an annual household income of ≤US $40,000. Participants were generally satisfied with the intervention, noting benefits including increased motivation to maintain PA beyond the duration of the intervention. Barriers included scheduling conflicts and inclement weather. Three theory-consistent themes framed participants’ evaluation of intervention success: accountability, accessibility, and ability. Accountability referred to support from the in-person group that facilitated motivation. Accessibility was described as the appreciation for resources provided by the intervention, including the provision of exercise equipment. Finally, participants discussed how their shared identity with other participants increased their sense of ability (or self-efficacy) to engage in PA. This was especially true for participants who described little to no engagement in PA prior to the intervention.

**Conclusions:**

To our knowledge, the MOMs intervention is the first of its kind: it delivers an in-person group PA intervention to high-risk mothers and women caregivers of children during their children’s sports practices. Findings from this study support the intervention’s delivery and integration to improve health behaviors, including PA, among mothers with intersectional and marginalized identities from the perspective of participants.

## Introduction

### Background

There has been an increase in the recognition of challenges mothers face in achieving recommended levels of physical activity (PA), underscoring the need for targeted interventions. Mothers are not engaging in enough moderate-to-vigorous-intensity PA to experience health benefits [1]. Instead, mothers experience significant declines in PA over time and report higher levels of physical inactivity, compared to nonparents [2,3]. Barriers to PA that are amplified for mothers include time constraints, childcare responsibilities, and inadequate support from spouse or partner [1,4,5]. A review of PA interventions for mothers suggested group-based programs and behavior change techniques (eg, goal-setting, self-monitoring, and problem-solving) are effective at increasing PA among mothers [6].

Black and Hispanic mothers are less likely to meet national PA guidelines than women from other racial and ethnic groups [7]. Notably, racial and ethnic disparities reflect the cumulative impact of structural inequities, as Black and Hispanic mothers are more likely to have lower socioeconomic status and less access to PA opportunities [8]. Moreover, and consistent with an intersectionality approach [9], the intersection of gender, socioeconomic status, and race creates unique challenges, which can make PA especially difficult for mothers with multiple marginalized identities. Prior interventions have not adequately addressed barriers that are unique to mothers with high-risk, including those from lower-income households and racial and ethnic minority groups [7]. These barriers include time limitations due to child commitments, childcare needs, and lack of access to PA opportunities [10-15].

### The Moms on the Move Intervention and Its Theoretical Framework

The Moms on the Move (MOMs) intervention is a PA intervention designed to comprehensively address PA barriers experienced by low-income Black and Hispanic mothers. The MOMs intervention builds on previous research that has focused on developing theory-based interventions that have demonstrated success at increasing PA among Black [16,17] and Hispanic or Latina [18-20] women. The behavioral theories underlying these interventions include Social Cognitive Theory (SCT) [21] and the Transtheoretical Model (TTM) [22]. The SCT suggests behaviors (including PA) can be better understood when considered within the context of personal characteristics, environmental factors, and the reciprocal interactions between the behavior, the person, and the environment [23,24]. The TTM was developed to recognize that people may vary in their readiness or motivation to change behavior, and that identifying this stage of change can be helpful when designing behavior interventions [22,25]. As a result, the MOMs intervention integrated intervention components that focus on theoretical constructs, including social support through the in-person group format of PA sessions, self-efficacy through the instruction of PA by a female fitness coach, and (in 2023) written materials tailored to the participant’s stage of change. A full list of theoretical concepts that guided the MOMs intervention and informed the present study are outlined in Multimedia Appendix 1.

What makes the MOMs intervention unique is that it is tailored specifically to Black and Hispanic low-income mothers and is delivered during children’s sports practices. Delivering the intervention in this context was intentional: It leveraged a setting where mothers were already present, helping to overcome barriers such as childcare needs, transportation, time constraints, and feelings of guilt associated with prioritizing self-care [26]. By integrating PA opportunities into their children’s schedules, the MOMs intervention allows mothers to engage in self-care without guilt while promoting consistent participation through the regularity of practices. Preliminary efficacy, as well as feasibility and acceptability, of the MOMs intervention has been established [27,28], providing support for this intervention. These findings are based on objective and self-report data, limiting an in-depth understanding of the participants’ perceptions of the intervention.

The MOMs intervention is a community-based program designed in partnership with a youth football and cheerleading organization. Community-based research [29] emphasizes the inclusion of community in the development of research. The MOMs intervention facilitated community involvement by soliciting community perspectives both during formative research, to understand barriers to engaging in healthy behaviors among Hispanic and Black mothers [30,31], and again when evaluating the intervention through exit interviews. Exit interviews are a qualitative analysis method that can facilitate process evaluation, specifically in the context of health-related intervention development [32-34].

### Current Study

We aimed to explore participants’ perceptions of the MOMs intervention using data from 2 rounds of exit interviews completed after 2 single-group pilot studies of the MOMs intervention. We were specifically interested in understanding participants’ engagement and satisfaction with the intervention, as well as their perceptions of benefits, barriers, and recommendations for future intervention development. The objective of our study was not only to better understand how to improve the MOMs intervention specifically, but also to guide program development for other mothers and women caregivers with multiple marginalized identities.

## Methods

### Study Design

This study used a qualitative analysis of exit interview data from participants who completed the MOMs pilot trials. To improve transparency and rigor, the Standards for Reporting Qualitative Research (SRQR) [35] and Template for Intervention Description and Replication (TIDieR) [36] guided information reported about the qualitative methods and intervention.

### Brief Overview of the MOMs Intervention

The MOMs intervention was delivered in partnership with a youth football and cheerleading organization in Providence, Rhode Island, with recruitment conducted during children’s football and cheerleading practices at the start of the season. Inclusion criteria included English-speaking women who had regular access to email and passed a screener to ensure they were not at increased risk for injury or harm by engaging in PA. Eligible participants had to identify as a primary caregiver of a child (age range of 6- to 13-years old) who was a member of one of the organization’s football teams or cheerleading squads (organized by age of the child). The choice to include all women caregivers, instead of exclusively mothers, was intentional to better adapt to the community-based setting and group-based nature of the program.

The first pilot study was completed in 2022; the second, in 2023, with insights from the first year informing intervention refinements in the second year. The primary intervention component during both years was in-person group PA sessions led by a female fitness instructor during children’s sports practices. The same female fitness instructor led the in-person group PA sessions both years (for 6 weeks in 2022 and 8 weeks in 2023). All in-person group PA sessions were an hour long and scheduled 3 times per week. Sessions were scheduled when at least 1, but up to 6, teams from the partner organization were practicing. Participants were invited and welcomed to attend all 3 sessions, regardless of whether their child was on the field and practicing. Child supervision, for younger children not participating on a football team or cheerleading squad, was offered. PA goal-setting calls and individually-tailored written materials (including tip sheets and stage-matched PA manuals) were added in 2023. Table 1 summarizes the MOMs interventions and their components.

Full information about the 2022 pilot trial, and preliminary outcomes, is described elsewhere [28]. Information about the MOMs pilot trial in 2023, including the procedures for exit interviews, is described in the ClinicalTrials.gov registration (NCT05461742); outcomes reporting the quantitative data are reported elsewhere [27].

**Table 1.** An Overview of the MOMs^a^ Interventions in 2022 and 2023 and and their components.

Intervention component	Year	
	2022	2023
Length of intervention	6 weeks	8 weeks
PA^b^ goal-setting calls	None	At the start of the intervention and midway through
Individually tailored written materials	None	Tip sheets and TTM^c^ stage-matched PA manuals were emailed weekly
Equipment	All participants were given a yoga mat. Other equipment, provided on field (eg, ropes, kettlebells, and resistance bands), was donated to the organization at the conclusion of the intervention.	All participants were given a workout bag with personal equipment (eg, small weights, a jump rope, and resistance bands), at the start of the intervention to keep.

a MOMs: Moms on the Move.

b PA: physical activity.

c TTM: Transtheoretical Model.

### Ethical Considerations

All study procedures were reviewed and approved by the Institutional Review Board of Brown University (protocol number: 2022003323). Participants provided informed written consent at the time of enrollment in the pilot study, including consent to complete an exit interview at the end of the intervention. Participants were compensated for completing assessments, with a total possible compensation of US $125 (in 2022) and US $130 (in 2023). All data were anonymized and deidentified for analysis.

### Recruitment for Exit Interviews

Convenience sampling was used to recruit participants for the exit interviews, with a desired sample size of 15 participants to capture a range of participant experiences. All participants who consented to participate in the MOMs pilot interventions (2022, n=26; 2023, n=35) were invited to complete an exit interview at the conclusion of the intervention (even if they did not attend any workout session). The 9 participants who participated in both interventions were invited to complete an exit interview at the end of each intervention. Participants who consented to the MOMs pilot interventions were not contacted for exit interviews if they had actively withdrawn from the study and asked not to be contacted (2022, n=4).

### Procedure for Exit Interviews

Exit interviews were completed by phone with a trained research assistant between October and November 2022 (for participants who completed the intervention in 2022) and between October and November 2023 (for participants who completed the intervention in 2023). A semistructured interview guide began with one broad question, “Overall, what did you think of the Moms on the Move intervention?” Follow-up questions focused on (1) exploring what participants liked and did not like about the intervention (eg, “Tell me what you liked about the intervention”), including barriers to participation (eg, “Can you think of anything else that we could have provided that would have been helpful in increasing your PA?”); (2) understanding participants’ perceptions of specific intervention components (eg, “What about the in-person PA did you find helpful in terms of increasing your PA?”); and (3) soliciting recommended changes for future intervention development (eg, “Do you have any other thoughts or comments about how we could improve the experience of participants in future studies?”). There were 13 follow-up questions for exit interviews conducted in 2022 and 16 follow-up questions for interviews conducted in 2023; the additional 3 questions added to the exit interviews were included to ask participants about additional intervention components (ie, goal-setting calls and written materials), as well as a question asking for participants who completed the intervention both years (2022 and 2023) to compare their experiences.

Interviews ranged between 7 minutes and 27 minutes and were audio recorded. Transcribed deidentified and deidentified interviews were imported to NVivo (Lumivero, version 14.23.3) for analysis.

### Data Analysis

A directed content analysis [37], informed by the SCT and TTM, was used. First, a codebook was developed using a deductive approach by identifying theory-related (Multimedia Appendix 1) and intervention-specific constructs (eg, social support and group component, respectively). The final codebook can be found in Multimedia Appendix 2. Transcribed interviews were coded by a team of coders (the first 3 authors: SGL, BO, and NR) immediately after codebook development. First, 2 interviews from 2023 were coded by all coders to establish consensus. Remaining interviews were then coded during team meetings by a pair of 2 coders, with a third coder observing and resolving any discrepancies. Pairs of coders were alternated to ensure equal participation across the 3 coders (SGL, BO, and NR). Next, codes were reviewed, and themes were identified by framing patterns from interviews within the SCT and TTM. All coders reviewed the themes to ensure the themes were consistent with the data. This approach was completed for the interviews from 2023 first, and then for the interviews from 2022. Given that coding began immediately after the development of a theory-informed codebook, our codes are considered predetermined [37]. However, we did not want to force the application of all codes; therefore, only those that were applied were reviewed for theme identification. Theory-informed themes based on coded data across both years were finalized during team meetings with the principal investigator (the last author, TvA).

## Results

### Participants

Nineteen participants were interviewed. Table 2 presents a full description of the sample. Participants were mostly Black or Hispanic. About half endorsed 12 or fewer years of education, and about two-fifths reported an annual household income less than or equal to US$ 40,000.

Participants primarily identified their relationship with the children participating in the sports practices as mothers, with 1 participant (Participant 4, 2022 and 2023) identifying as a grandparent.

**Table 2.** Participant demographic characteristics.

Demographics	Values (N=19)
Age^a^ (years), mean (SD)	38.8 (8.5)
Total no.of children <18 years old living at home^b^, mean (SD)	2.3 (1.3)
Years participated, n (%)	
2022	6 (32)
2023	10 (53)
2022 and 2023	3 (16)
Marital status^c^, n (%)	
Single^c^	12 (63)
Partnered^d^	6 (32)
Missing	1 (5)
Education level, n (%)	
≤12 years	10 (53)
>12 years	5 (26)
Missing	4 (21)
Race, n (%)	
Black or African American	6 (32)
Multiracial	6 (32)
White	2 (11)
Missing	5 (26)
Ethnicity, n (%)	
Non-Hispanic or Latina	14 (74)
Hispanic or Latina	5 (26)
Income, n (%)	
≤US $40,000	8 (42)
> US $40,000	7 (37)
Missing	4 (21)
Family history, n (%)	
First generation	1 (5)
Second generation	6 (32)
Third generation	7 (37)
Missing	5 (26)

a Range: 30‐62.

b Range: 0‐5.

c Single includes single (n=7), never married (n=3), widowed (n=1), and separated (n=1).

d Partnered includes never married (n=3), living with partner but not married (n=1), married (n=1), and in a relationship but not living together (n=1).

### Findings

We sought to examine participants’ perceptions of engagement and satisfaction with the MOMs intervention, as well as benefits, barriers, and recommendations. Overall, participants were generally satisfied with the MOMs intervention. More specifically, participants described the MOMs intervention as “motivating,” “comfortable,” “good,” and “helpful.” Self-reported benefits of the intervention included improved overall health and increased motivation to engage in PA. While barriers were still acknowledged, including inclement weather and guilt associated with prioritizing care for self, the consensus was that the intervention should be continued. Four theory-informed themes were identified. The first centers around takeaways from the interventions. The next 3 identify the essential qualities associated with the perceived success of the intervention: accountability, ability, and accessibility. Exemplar quotes are included with a unique participant identifier, and the years participants completed the intervention.

#### Theme 1: Takeaways of the Intervention: Benefits and Recommendations

Participants identified various benefits of the intervention. The most robustly described benefits were on the individual level and were associated with key constructs of the SCT and TTM. Participants noted increases in their self-efficacy to engage in PA, their enjoyment when engaging in PA, and their motivation to engage in PA beyond the group sessions (even after the intervention was completed). For example, 1 participant who participated both years shared that she was:

...actually pretty good still with [the ladder exercise] this year, like with the footwork. So, I enjoyed that.

[Participant 4, 2022 and 2023]

Another participant described plans to continue exercise once the intervention ended:

...probably more at home because I know [the sessions are going to] end soon.

[Participant 13, 2023]

Beyond these PA-related changes, participants also noted improved mental and physical health. Multiple participants reported improved energy levels and sleep. They also reported improved mental health, for example,

[PA] helps me mentally feel better and it improves my mood.

[Participant 14, 2023]

When explicitly asked about stress, there were mixed responses from participants: A few participants said that they noticed less stress when engaging in the intervention, others said they did not notice anything, and some said stress was something that was a barrier to PA, for example:

...like the stress makes me… not want to do anything either.

[Participant 8, 2022]

These individual-level benefits were paired with family-level benefits. Specifically, participants described sharing the knowledge and skills gained from the intervention with their families, offering opportunities to increase PA as well as to connect. Participants talked about how the intervention improved their bond with their child who was on the sports team. For example, 1 participant shared a newfound understanding of their child’s sport as the intervention:

...made me look at how hard my son was working because the exercises were, some were really hard and you know, you really have to put in work. So, it made me have a whole different outlook on, you know, his practices and how much work he puts in daily.

[Participant 16, 2023].

Bonds were also improved through opportunities to engage in PA together, even after the intervention ended, as shared by Participant 6 (2022):

I feel like it’s a household thing now, like even just trickled over and even like we’re just challenging each other, and he [the participant’s son] is like, ‘Come on, Mom, we gotta work out. We gotta stretch.’ So, like I appreciate it now because I feel like it became something that now we’re both kind of like just into it and embedded in it.

While participants reported various benefits, they also identified barriers to participation, including scheduling and time conflicts, inclement weather, pain and physical symptoms (eg, shortness of breath), low motivation, low group attendance, guilt associated with prioritizing care for self, and competing demands (eg, taking care of other children not practicing, preferring to watch their child practice, and work). Interestingly, individual-level barriers (eg, low motivation and laziness) came up more for participants in 2022, which might be because the 2023 intervention had more components (including goal-setting calls) that supported facilitated behavior change.

Recommendations solicited from participants included a combination of amplifying benefits and mitigating barriers. For example, participants appreciated the in-person group sessions but also suggested that sessions be offered virtually as well, to accommodate for scheduling conflicts or inclement weather. A full list of the recommendations suggested by participants is included in Table 3.

**Table 3.** Summary of recommendations suggested by participants during exit interviews*.*

Intervention component	Years	Recommendations
Goal setting calls^a^	2022	Participants were in favor of goal-setting calls. They noted that these calls could be via email or text check-ins, vs phone. They also recommended check-ins beyond the length of the intervention.
Goal setting calls^a^	2023	Participants suggested increasing the frequency of the goal-setting calls to a weekly basis and also recommended check-ins beyond the length of the intervention for continued accountability.
Individually tailored materials^a^	2022	Participants agreed with the plan for individually-tailored materials to be introduced in 2023. When asked about suggestions for content, they noted: nutrition and meal plans, stretches, muscle strengthening, and suggestions for brief bouts (15‐30 minutes), and reminders about proper form.
Individually tailored materials^a^	2023	Participants recommended the intervention could provide more personalized materials, including workouts to complete at home, and specific topics, such as nutrition, breathing, and weightlifting.
Gender of group	2022 and 2023	Participants generally preferred having the gender of the group be women only; however, some noted they did not mind men participating.
Gender of fitness instructor	2022 and 2023	Participants generally preferred having a woman fitness instructor; however, several did note that the gender of the fitness instructor did not matter.
Group component	2022 and 2023	Participants generally liked the in-person group component. They were enthusiastic to increase participation in the group.
Content of the intervention	2022 and 2023	A few participants indicated interest in expanding the content of the intervention to cover other healthy behaviors, namely healthy eating.
Duration and logistics	2022 and 2023	A few participants requested that the intervention length be extended beyond football season. Participants also recommended virtual options for sessions, especially when there were disruptions due to schedule conflict or weather.
Incentives	2022 and 2023	Participants offered suggestions to increase incentives, including the distribution of merchandise promoting a sense of camaraderie.
PA^b^	2022 and 2023	Participants recommended different types of PA, as well as supplemental opportunities for PA (eg, membership to a gym). Participants in 2022 reported that some exercises (eg, jumping jacks) were too challenging; as a result, modifications were added to the PA intervention in 2023.
Assessments	2022 and 2023	Participants requested for assessments to be shorter and less repetitive; as a result, the assessment midway through the intervention was removed in 2023. When asked about preference for completing assessments, many requested a preference for internet- or phone-based assessments.

a Note that these components were not included in the 2022 intervention. For this year, the recommendations listed summarize participants’ responses to whether they would like these components added, and if so, what recommendations they had for these components.

b PA: physical activity.

Support to incorporate these recommendations is evident in participants’ comparisons of the interventions (ie, 2022 vs 2023). For example, some participants in 2022 described the exercises as being too difficult. Participant 7 (2022) noted that the jumping jacks were “a little hard for moms.” As a result, the exercises were changed in 2023 (eg, including weights and resistance training), and modifications to exercises were introduced. Thus, participants in 2023 were better able to participate regardless of their fitness level and could benefit from learning a variety of exercises. As noted by participant 6 (2022 and 2023) when asked about what she liked about the intervention:

The weights and resistance bands – just to kind of change it up. So, you get like that slow-to-moderate. So, it was kind of like a switch up with the weights and all that good stuff incorporated with the cardio. So, I like that.

While not a component of the intervention, assessments were also included in the list of recommendations in Table 2, given that many participants spontaneously described these assessments as a part of the intervention that could be improved. Participants described the assessments as “repetitive” (Participant 6, 2022 and 2023) and “tedious” (Participant 3, 2022). Participants indicated internet- or phone-based assessments would be preferred, suggesting that it is not necessarily the assessment itself that needs improvement, but how it can be administered.

#### Theme 2: Accountability

One of the essential qualities of the intervention was accountability. Accountability was facilitated by the group and in-person nature of the intervention, goal-setting phone calls from the intervention team, and having a female fitness instructor who was part of the organization’s community. Participants tended to be most appreciative of the ways the MOMs intervention facilitated accountability to participate in the intervention and engage in PA, more generally.

Accountability was facilitated by the group and in-person nature of the intervention in 3 ways. First, participants appreciated the support they received from other participants to engage in the intervention. One participant summarized that the participants

...pushed each other, you know, we laughed, we had a good time. We would like to be together. I think we worked well together.

[Participant 14, 2023]

Second, participants felt a sense of responsibility toward their peers, which often motivated them to be engaged with the intervention. For example, Participant 19 (2023) stated that she felt the group encouraged her to learn a new exercise:

The way we pushed and encouraged each other. That was helpful. Like, in the beginning, when I first started out with the ropes, I felt like I was gonna die. And I swear, by the end, I was like, I mastered it. You know?

Third, participants developed a collective efficacy, or a sense of accomplishment on a group-level, and group cohesion; this was perceived as a sense of affinity, for example:

...[the participants] are trying to stay active, just like me.

[Participant 3, 2022]

This perceived affinity acknowledged how participants experienced their personhood in the context of a group narrative, and how this experience was conducive to PA engagement.

Accountability was also facilitated with goal-setting phone calls from the intervention team. Specifically, the goal-setting calls:

...just kind of kept you on track. It kept you mindful of what your goals were, what you wanted to improve. So again, like that accountability piece.

[Participant 14, 2023]

In 2022, the intervention did not include a goal-setting call component; however, participants were asked if such a component would be helpful. Participants responded that they liked the idea; thus, goal-setting calls were introduced in 2023. In 2023, participants described these calls as helpful in keeping participants accountable to engage in the intervention and in PA more generally.

Having a shared identity with the fitness instructor also facilitated accountability. In both 2022 and 2023, the fitness instructor who led the workout sessions was female and had a child who played football for the same organization. Having a female fitness instructor

...makes it seem like it’s a complete sisterhood. There’s no guys almost like young kids and it’s like no boys allowed. So that was good.

[Participant 15, 2023]

All participants who completed the exit interviews were appreciative of the fitness instructor and how her skill, style, and affiliation with the organization were appropriate and encouraging. The fitness instructor played a unique role by being someone who was a part of the community, yet separated by her role as an instructor. She was regarded as “very knowledgeable” [Participant 14, 2023].

While participants preferred to have a female fitness instructor, some reported that they would not mind having a male fitness instructor.

Two less frequent, but still relevant, sources of accountability were self-accountability and family accountability. The commitment to staying engaged in PA beyond the intervention was described as dependent on the individual-level initiative to stay active. For example:

I like the tools that she gave us, so I think it’s just up to the individual to like, you know, get up, be motivated, and want to do more. Like just this morning, I know it’s cold, but I gotta get my walk in because it’s going to rain sometime today and, if I’m not walking, I’m doing physical [activity], like raking my leaves and I was over 12,000 steps yesterday.

[Participant 4, 2022 and 2023]

Additionally, participants cited support from family as another accountability source. Family (including, but not limited to, the children who were practicing at the field while participants were engaging in the intervention) was described as a source of motivation to engage in PA, as well as a source of support to engage in continued PA. One participant described how

...my son did want me to get out and exercise, so he actually would be running, doing his laps and seeing me doing it. It was so exciting for him as well.

[Participant 16, 2023]

Of note, avenues to achieve accountability seemingly increased from 2022 to 2023, primarily through opportunities to engage in goal-setting calls and an increased sense of community as a result of another year of engaging in the intervention together. However, the greatest change noted by Participant 6 (who completed the intervention in 2022 and 2023) was in attendance, and how having more people led to greater motivation to engage in the intervention:

[It] was great just having more moms to kind of work out with this year. I mean, I feel like last year it was the same because we had like equipment and things like that – and I always think that that is super helpful – [but…] I love that there was more participation, I would say, this year.

#### Theme 3: Ability

Ability (including narratives surrounding self-efficacy, confidence, and motivation) to engage in PA was a factor that shaped participants’ experiences with the intervention. Participant 16 (2023) shared that joining the intervention with other participants:

...gave me, like, the confidence to like exercise and you know, being around the other [moms]. You know, usually when I would go into, like, the YMCA, there’d be a lot of younger girls and you know, they have moving, moving, moving, but it just gave me the confidence to keep pushing and continue.

Part of what made the group effective was also the shared identities of the participants. All the participants interviewed also appreciated having the ability to connect with other mothers and women caregivers in the group, often noting shared identities, including being a football mom. This appeared to be especially true for participants who started the intervention with lower, or no, PA; these participants tended to want more overlapping identities with other members of the intervention (eg, women-only groups led by a woman instructor). For example, one participant shared:

...women felt comfortable with the female [instructor]. Especially those that are just beginning or might need some support and like kind of figuring out their body and their ability to move. I think it’s just more comfortable to have a female there to support.

[Participant 14, 2023]

Similarly, another participant described an appreciation for the intervention providing space for

[Older] women around my age. And no guys, I like that… Now that I’ve been doing [exercises] and I feel confident, it’ll be fine [to include men in the sessions]. But initially, yeah, you know.

[Participant 16, 2023]

Related to this, engaging in a shared activity with individuals who share an identity was key to changes in PA self-efficacy. As shared by another participant (Participant 3, 2022),

I didn’t feel like I was by myself working out. I felt like I was with other people that were doing the same thing I was doing, so it helped my self-esteem a little bit, my self confidence in me working out.

On the other hand, the decline in attendance toward the end of the intervention led to a decrease in motivation for this participant; she

...did them [the exercises] all the way until like the end and then like once I stopped seeing everybody going over there, then I stopped going.

[Participant 3, 2022]

Another way ability showed up was when participants discussed their readiness to change their behavior. Most participants started in the earlier stages of change as indicated by their responses in exit interviews and moved toward action and maintenance. For one participant, the intervention

...kind of started the ball rolling, so now I’m encouraged to keep going.

[Participant 14, 2023]

However, this was not the case for all participants; some described a more contemplative state about continuing PA beyond the intervention, for example, Participant 8 (2022) stated, “I’m slowly starting”

Regardless of the ability to adequately capture stages of change in interview responses, there was a general sense that participants were moving across stages of change toward engaging in PA, which was most evident when participants were asked whether and how they would continue their PA at the end of the intervention. Namely, some talked about continuing to use the equipment, to continue following the structure (eg, engaging in PA during their children’s other sports such as at basketball practice), and, to continue to seek accountability (eg, finding a buddy to engage in PA with, whether another mom from the group or a family member or friend). Additionally, participants, including Participant 4 (2022 and 2023), shared their plans to continue to be active during her child’s sports practices: for example, “take a few people and we would walk around the track or something like that.”

#### Theme 4: Accessibility

Participants shared that the intervention made PA accessible. Accessibility was evident in participants’ descriptions of the intervention, including adjectives like “opportunity,” “flexibility,” and even “comfort.” This theme was developed as a result of observed patterns in conversations about specific intervention components, including: “the exercises that I learned from [the fitness instructor]” (Participant 6, 2022 and 2023); the exercise equipment that the intervention team provided, for example,“the bands and the dumbbells and the jump rope” (Participant 18, 2023); scheduling, for example, “being able to be on the side of the field while working out while he’s on the other side was amazing” (Participant 2, 2022); and child supervision provided by the intervention team for participants who have younger children not practicing.

Regarding the exercises taught by the fitness instructor, participants appreciated learning varied ways to use the equipment and becoming more knowledgeable about different types of exercises. In 2022, some participants described being motivated to buy equipment themselves to stay active outside of sessions. For example, Participant 9 (2022) shared she would benefit from

...a take-home kind of kit, whether it’s like a jump rope or weights, or even like a, you know, exercises to do at home if we weren’t able to get, you know, do it at the field.

Providing equipment for participants to take home and teaching proper use, with variations and modifications for women with different fitness levels, helped to increase accessibility for the mothers and women caretakers who participated in 2023, even beyond the PA sessions as illustrated by a quote from Participant 3 (2022 and 2023):

I still use my equipment. Like, it’s actually perfect. Me and my son use the equipment.

The intervention was delivered during children’s sports practices and thus participants noted that it was convenient for them as they did not have to carve out time outside of their routine. However, children of different age groups sometimes practiced on different days or at another location, leading the in-person group PA sessions to be more convenient for some participants than others. Due to the pilot intervention’s limited funding, sessions were limited to 3 times per week at 1 of the 2 practice fields; thus, attending was sometimes not feasible for participants with children practicing on different days or at the other field. One participant who had 2 children on different teams described:

...one [of my children] was at the other field and my son was at the other field. So, it was kind of hard for me to be at two fields at the same time.

[Participant 17, 2023]

Moreover, cancelations (eg, as a result of inclement weather) made by the organization also disrupted participation and engagement; for example, there were days in which:

[…] they’ll cancel practice at like the last minute and you got to switch around everything because, you know, that would have been another day to be in person working out.

[Participant 15, 2023]

The individually-tailored materials present an interesting story about accessibility. While the participants in 2022 did not have individually-tailored materials, they expressed interest in these materials when asked during their exit interviews. Some even offered ideas of what those materials could include, with suggestions of nutrition, meal plans, and examples of stretches and small bouts of exercise (eg, described by Participant 5, who participated in 2022 only, as “things that are like short, more realistic, like say if it’s like 30-min or 15-min tidbits [of PA]”).

However, the participants in 2023 did not really engage with the individually tailored materials. Of note, the individually tailored materials in 2023 were strictly PA focused and did not include material on additional topics of interest noted by participants in 2022.

## Discussion

### Brief Summary

This study examined the qualitative data from 2 exit interviews completed after each year the MOMs intervention was piloted to explore participants’ perceptions of the program and guide future intervention development. For the most part, the MOMs intervention was described as a helpful way to increase PA for this sample of predominantly Black and Hispanic mothers and women caretakers with low income because of its convenience and components, embedded within a community context. Findings were aligned with the SCT and TTM, given that the most salient characteristics of the intervention were associated with social support (described as accountability), self-efficacy (described as ability), and environmental facilitators (described as accessibility). Coupled with results from a quantitative analysis that found that self-efficacy and self-reported moderate- to vigorous-intensity PA increased among MOMs participants [28] and an exploration of the feasibility and acceptability of the MOMs intervention [27], these findings support the continued delivery of the program, as well as its ability to be expanded to better support high-risk mothers and women caregivers.

### Principal Findings and Comparison to Prior Work

Health behavior change theories, including SCT [24] and TTM [22], identify social influences on behavior change, for example, the impact of the social context and of helping relationships. Data from this study add to and extend theory in 3 ways. First, given that participants appreciated a sense of shared identity (or ability) with those in the PA group, it appears that it is not only about having relationships with other people but how relationships transform motivation that is relevant to health behavior change. The support received from a group of similar others can be characterized as a distinct motivating factor that might impact behavior differently. For example, while stages of behavior change are typically thought of as being linear (ie, one stage leads to the next) and circular (eg, people who are in a maintenance stage of change can become precontemplative again) [22], the fluidity of these stages was notable for participants. Additionally, given that this group is embedded within the community, there are opportunities for this intervention to have a sustained impact. For example, participants have the opportunity to engage in PA with group members outside the sessions during other sports practices, or even other sports seasons. Second, given that participants noted how their engagement in the intervention changed how their children viewed their mother’s behavior and the importance of healthy behaviors, it seems that modeling, often understood as identification with a role model [38], might also extend to identification as a role model (ie, recognizing that your behavior has an impact on another).

Interestingly, motivation and self-efficacy (as well as a number of other individual-level factors) were described as outcomes of participating in the MOMs intervention, rather than simply a predictor of engaging in health behaviors. In other words, participants began engaging in behavior change because an opportunity was presented to them, and they appreciated the group nature of it, specifically. Then, after beginning to engage in the intervention, they noticed an increase in motivation. The notion that motivation can come from relationships, rather than oneself, has been identified in relationship science, for example, interdependence theory [39,40], but has infrequently been extended beyond nonromantic contexts. Future studies may benefit from integrating interdependence theory with health behavior change theories, including those presented in this study (the SCT and TTM) as well as others that are relevant to expand our understanding of how social relationships transform motivation to engage in behavior change. In particular, self-determination theory [41] may be important to consider, given how concepts related to the 3 basic psychological needs (competence, autonomy, and relatedness), intrinsic and extrinsic motivation, and motivational climate showed up in the data.

Finally, these participants did not need more internal motivation to get their PA started; they needed opportunity presented by a supportive environment. Opportunity connects to the theme of accessibility. Early health behavior change theory literature originated from understanding preventive behavior [42]; however, increasing healthy behaviors may operate differently (and this could be why some a priori codes were not present, eg, stimulus control). Aligned with the SCT and TTM [22,24] and a growing body of literature on PA specifically [43,44], our findings suggest that creating environments in which healthy lifestyle behaviors, including PA, can occur is an efficacious strategy to facilitate behavior change among underactive populations. In particular, children’s sports practices are a good context for health behavior interventions for mothers and women caretakers with marginalized and intersectional identities.

### Implications

Future research focused on developing the MOMs intervention can adopt stronger study designs (eg, a fully powered randomized controlled trial) to test the effects of the intervention for high-risk mothers and women caregivers. Recommendations for interventions are drawn from combining themes identified in these exit interviews with participant suggestions (Table 2) and literature on health behavior change. First, given the helpfulness of having accountability and the known influence of helping behaviors [22], it may be beneficial to implement a buddy system (eg, where participants who have completed the intervention at least once can become role models for others who are newer to the intervention). Adding to this social component, opportunities to increase group cohesion and collectiveness may be facilitated in a number of ways, including having ice breakers at the beginning of each session to facilitate bonding and a sense of connection to increase opportunities for mothers to connect about shared identities. Given that bonding with family was also a benefit, it may be important to consider opportunities for introducing shared activities for mothers and their children during practices (eg, completing warmups together). Finally, facilitating more accessibility within the environment could be made possible with opportunities to engage in the intervention either remotely or asynchronously; this would also help reduce disruptions in case of weather disruptions and other schedule changes. This can be made possible through the use of online platforms and technology, as recommended by the participants, and a growing body of research has already begun exploring this in the context of lifestyle interventions [45], especially those developed for women from minoritized backgrounds [46,47]. It is possible that the inclusion of both in-person and technology-based intervention components may allow for increased engagement and improved outcomes.

One of the least liked aspects of the intervention was the assessment. Until this program can be integrated within the community, assessments are necessary for the scientific process of intervention development and evaluation. Therefore, while they cannot be removed at this stage of intervention development, changes can be made to make the assessments less burdensome. The redundancy may be eliminated by gathering data during intervention delivery, for example, during goal-setting calls; this way, the data can be collected in a useful manner for the participant. Incentives for assessment completion can also be adjusted to better reflect what would be appropriate for the moms.

### Limitations

While this study allowed us to gain an in-depth understanding of what worked for participants in the MOMs pilot studies, there are limitations. First, the study combines distinct cohorts with different intervention components and study procedures. Nonetheless, our results suggest that there were overlaps in benefits and recommendations that may help to inform future PA intervention efforts. Second, only 31% (19/61) of participants (19 out of a total of 61 participants enrolled in the 2 pilot studies) completed an exit interview. It is likely that there is a sampling bias in that participants who agreed to complete the exit interview may have had more helpful and positive things to say. For example, while participants overall regarded the fitness instructor in a positive light during the interviews, we know from recruitment efforts that there were participants who declined to participate in the research study upon discovering the fitness instructor’s identity. Future interventions may benefit from offering anonymous surveys at the end for those who did not complete an exit interview. Additionally, because we intentionally developed and tested the MOMs intervention for lower-income Black and Hispanic mothers, there may be limits to generalizability. All our participants were also English-speaking and from a single community organization. It is possible that the sociodemographic factors and history of this organization impacted the context in which the intervention occurred. Finally, while the intervention was designed to focus on mothers whose children were already enrolled in sports to address the barrier of time, this limits the ability to engage mothers whose children are not enrolled. Future interventions may benefit from additional ways to include minoritized mothers, for example, including PA sessions during other children’s activities.

### Conclusion

Results from our qualitative analysis of exit interviews from a community-based PA intervention delivered to high-risk mothers and women caregivers during children’s sports practices suggest participants endorsed enjoyable experiences and perceived positive outcomes due to their engagement with the intervention. These findings align with quantitative data on the MOMs intervention [27,28], and support the continued development and testing of PA interventions delivered during children’s sports practices to address the unique needs of this high-risk population. In sum, low-income Black and Hispanic mothers and women caregivers may benefit from programs that include components focused on facilitating accountability, tailoring to ability, and increasing accessibility.

## Supplementary material

10.2196/87120 Multimedia Appendix 1 Theoretical concepts that informed this study, and their definitions.

10.2196/87120 Multimedia Appendix 2 Codebook.

## Abbreviations

MOMs: Moms on the Move
PA: physical activity
SCT: Social Cognitive Theory
SRQR: Standards for Reporting Qualitative Research
TIDieR: Template for Intervention Description and Replication
TTM: Transtheoretical Model
