# Supplementary material for: A Physical Activity Intervention for High-Risk Mothers Whose Children Play Sports: Qualitative Analysis of Exit Interviews From Two Pilot Studies
Source: JMIR Form Res. 2026 Oct 8;10:e87120. doi: 10.2196/87120 (PMC13649072; doi:10.2196/87120)
Supplement: Supplementary file 1 [file formative-v10-e87120-s001.docx]

**APPENDIX 1**

**Theoretical Concepts that Informed the Present Study, and their Definitions.**

| **Theory and Theoretical Construct** [1,2] | **Definition** |
| --- | --- |
| SCT |  |
| - *Social Support* | Support or encouragement from another (e.g., friends and family) to engage in a behavior |
| - *Enjoyment* | The extent to which a behavior is experienced as pleasurable and positive |
| - *Self-Efficacy* | One’s confidence in their ability to perform a behavior |
| - *Expectations* | Anticipated outcomes of a behavior |
| TTM |  |
| - *Pre-Contemplation* | A stage of behavior change characterized by no intention to take action |
| - *Contemplation* | A stage of behavior change characterized by intention to take action |
| - *Preparation* | A stage of behavior change characterized by intention to take immediate action and steps toward behavior change |
| - *Action* | A stage of behavior change characterized by overt behavior for up to six months |
| - *Maintenance* | A stage of behavior change characterized by overt behavior for more than six months |
| - *Decisional Balance* | A cognitive process that involves weighing the benefits (pros) and consequences (cons) of changing their behavior |
| - *Processes of Change* | Behavioral and cognitive processes that help people progress through stages of change, including increasing knowledge and caring about the consequences of their behavior on others |

*Note:* SCT (Social Cognitive Theory); Transtheoretical Model (TTM).

**References**

1.	Marcus BH, Pekmezi D. Motivating people to be physically active. Human Kinetics; 2024. ISBN:1-7182-1700-5

2.	Glanz K. Theory at a glance: A guide for health promotion practice. US Department of Health and Human Services, Public Health Service, National …; 1997.
