# Supplementary material for: A Physical Activity Intervention for High-Risk Mothers Whose Children Play Sports: Qualitative Analysis of Exit Interviews From Two Pilot Studies
Source: JMIR Form Res. 2026 Oct 8;10:e87120. doi: 10.2196/87120 (PMC13649072; doi:10.2196/87120)
Supplement: Supplementary file 2 [file formative-v10-e87120-s002.docx]

**APPENDIX 2**

**Codebook**

**Social Cognitive Theory Constructs**

**Social Support (+/**-**):** support, or encouragement, to engage in either (a) physical activity (PA) and/or (b) the intervention from (1) family, (2) friends, (3) the group, and/or (4) the coach.

**Enjoyment (+/**-**):** satisfaction from the (a) PA and/or (b) the intervention (include ***Intervention Qualifier***); in other words, the extent to which the intervention was experienced as pleasurable and positive.

**Self-Efficacy (+/-):** confidence in the ability to participate in (a) PA or (b) the intervention.

**Outcome Expectations:** beliefs regarding the effects of engagement in the intervention; this might come up as expectations participants had before or during the intervention, or for beliefs about maintaining PA beyond (or after) the end of the intervention.

**Transtheoretical Model Constructs**

**Stage of Change:** any time participants refer to their readiness to engage in (a) PA and/or (b) the intervention. Qualify the stage of change, wherever possible: (1) pre-contemplation, (2) contemplation, (3) preparation, (4) action, (5) maintenance.

**Decisional Balance (Pro/Con)**: any time participants refer to the pros and cons of participating in the intervention, where pro describes the benefit of engaging in (a) PA and/or (b) the intervention and con refers to the disadvantages/costs of engagement.

**Processes of Change:** any time participants reference what helped them make and maintain change. Qualify the process of change, wherever possible: (1) consciousness-raising (increasing awareness); (2) dramatic relief (feelings related to unhealthy behaviors); (3) self-reevaluation (realizing PA is an important part of personal goals/identity); (4) environmental reevaluation (realizing how unhealthy behaviors, e.g., not engaging in PA, impact others); (5) social liberation (realizing society is supportive of PA); (6) self-liberation (believing in one’s ability to change); (7) helping relationships (identifying people who are supportive of change); (8) counterconditioning (using substitutes); (9) reinforcement management (increasing rewards from PA); (10) stimulus control (managing environment using reminders and cues).

**Intervention Elements**

**Barriers:** anything that prohibited or limited (1) PA, (2) participation in the intervention, and/or (3) other.

**Facilitators:** anything that enabled or increased participation in (1) PA, (2) participation in the intervention, and/or (3) other.

**Recommended Changes:** any mention of changes that participants recommended. Where possible, qualify the part of the intervention that was changed: (1) group component, (2) individually tailored materials, (3) assessments, (4) program logistics: (a) duration, (b) frequency, (c) intensity, (5) content of program: (a) specific exercises and/or (b) equipment; and/or (6) other.

**Changes Experienced (2022 vs 2023):** any mention of changes to the intervention that participants noted (this is exclusive to the participants who completed interviews in 2022 and 2023).

**Intervention Effects:** any time a participant describes the impact of the intervention. Qualify the domain of the impact, where possible: (1) PA, (2) health and wellbeing, including (a) physical and/or (b) mental health; (3) family; (4) financial situation; (5) integration within the community or the organization; (6) community-building; and/or (7) other.

**Stress and PA:** any time a participant talks about the relationship between stress (and/or stress management) and physical activity. (Note: This code was added because the relationship between stress and PA was explicitly asked about in the 2022 exit interviews.)

**Intervention Qualifier**

Wherever possible, qualify the part of the program the participant is referencing.

**Intervention-specific** qualifiers include: (1) group component, (2) individually tailored materials, (3) fitness instructor; (4) goal setting calls; and (5) program logistics, including: (a) duration, (b) frequency, (c) intensity, and (d) content of program (specific exercises and/or equipment).

**Assessment** qualifiers include any time a participant references the assessments or study process as separate from the intervention/program.

**Incentive** qualifiers include any time participants referenced their motivation from the study team to participate.

**Recruitment** qualifiers include any time a participant referenced recruitment efforts.

**Physical activity** qualifiers include any time a participant references the physical activity as separate from the intervention/program.

**Other:** any time a participant mentions something that does not necessarily fall into another code but is (1) relevant to the research questions and/or (2) interesting, different, or unique.
